# CRISPR-mediated detection of *Pneumocystis* transcripts in bronchoalveolar, oropharyngeal, and serum specimens for *Pneumocystis* pneumonia diagnosis

**DOI:** 10.1172/JCI177241

**Published:** 2025-03-03

**Authors:** Brady M. Youngquist, Ayanda Trevor Mnguni, Dora Pungan, Rachel PJ Lai, Guixiang Dai, Chun Fai Ng, Amy Samson, Yasmean Abdelgaliel, Christopher J. Lyon, Bo Ning, Shahid Husain, Sean Wasserman, Jay K. Kolls, Tony Y. Hu

**Affiliations:** 1 Center for Cellular and Molecular Diagnostics, Department of Biochemistry and Molecular Biology, School of Medicine, Tulane University, New Orleans, Louisiana, USA.; 2 Centre for Infectious Diseases Research in Africa, University of Cape Town, Cape Town, South Africa,; 3 Africa Mycology Unit, University of Cape Town, Cape Town, South Africa and Department of Internal Medicine, University of Cape Town, Cape Town, South Africa.; 4 Department of Internal Medicine, University of Stellenbosch, Stellenbosch, South Africa.; 5 Center for Translational Research in Infection and Inflammation, School of Medicine, Tulane University, New Orleans, Louisiana, USA.; 6 Department of Infectious Disease, Imperial College London, London, United Kingdom.; 7 Multi-Organ Transplant Program, Division of Infectious Diseases, Department of Medicine, University Health Network/ University of Toronto, Toronto, Ontario, Canada.; 8 St. George’s University, London, United Kingdom.

**Keywords:** Infectious disease, Pulmonology, Diagnostics, Mitochondria, Mouse models

## Abstract

**BACKGROUND:**

*Pneumocystis jirovecii* pneumonia (PCP) is a leading cause of fungal pneumonia, but its diagnosis primarily relies on invasive bronchoalveolar lavage (BAL) specimens that are difficult to obtain. Oropharyngeal swabs and serum could improve the PCP diagnostic workflow, and we hypothesized that CRISPR could enhance assay sensitivity to allow robust *P*. *jirovecii* diagnosis using swabs and serum. Herein, we describe the development of an ultrasensitive RT-PCR–coupled CRISPR assay with high active-infection specificity in infant swabs and adult BAL and serum.

**METHODS:**

Mouse analyses employed an RT-PCR CRISPR assay to analyze *P*. *murina* transcripts in WT and *Rag2^–/–^* mouse lung RNA, BAL, and serum at 2-, 4-, and 6-weeks after infection. Human studies used an optimized RT-PCR CRISPR assay to detect *P*. *jirovecii* transcripts in infant oropharyngeal swab samples, adult serum, and adult BAL specimens from patients who were infected with *P*. *jirovecii* and those who were not.

**RESULTS:**

The *P*. *murina* assays sensitively detected *Pneumocystis* RNA in the serum of infected mice throughout infection. Oropharyngeal swab CRISPR assay results identified infants infected with *P*. *jirovecii* with greater sensitivity (96.3% versus 66.7%) and specificity (100% versus 90.6%) than RT-qPCR compared with mitochondrial large subunit rRNA gene (*mtLSU*) standard marker, and CRISPR results achieved higher sensitivity than RT-qPCR results (93.3% versus 26.7%) in adult serum specimens.

**CONCLUSION:**

Since swabs are routinely collected in pediatric patients with pneumonia and serum is easier to obtain than BAL, this assay approach could improve the accuracy and timing of pediatric and adult *Pneumocystis* diagnosis by achieving specificity for active infection and potentially avoiding the requirement for BAL specimens.

**FUNDING:**

The work was supported by the NIH (R01AI120033), NHLBI (R35HL139930), the Louisiana Board of Regents Endowed Chairs for Eminent Scholars program, and by research funding provided by National Institute of Allergy and Infectious Diseases (NIAID) (R01AI144168, R01AI175618, R01AI173021). This research was also funded by the NIHR (project 134342) using UK aid from the UK government to support global health research.

## Abstract 2

This paper describes novel-based RNA detection tools to detect Pneumocystis jirovecii pneumonia.

## Introduction

Molecular epidemiology evidence indicates that *Pneumocystis jirovecii* pneumonia (PCP) is the leading cause of fungal pneumonia in HIV-negative infants under 2 years old (1, 2), but PCP is also clinically relevant in adults and children with immunodeficiencies or who are receiving immunosuppressive regimens (2). *P*. *jirovecii* infections that cause severe disease and require mechanical ventilation can have mortality rates of 20%–25% (3). Rapid PCP diagnosis is required for effective therapeutic intervention, but current diagnostic tests require an invasive bronchoalveolar lavage (BAL) procedure to obtain diagnostic specimens, which can delay diagnosis (4, 5), and use Grocott methenamine silver (GMS) or immunofluorescent staining methods or PCR of *P*. *jirovecii*-specific genomic DNA to detect *P*. *jirovecii* infection (6). However, there is evidence that an organism-specific diagnostic that uses minimally invasive or noninvasive samples is needed to improve diagnosis (7–9). Direct fluorescent antibody staining of induced and expectorated sputum has variable sensitivity for *P*. *jirovecii* and is primarily useful in patients who are HIV-positive, who have higher *P*. *jirovecii* burdens than other patients with PCP (10), while a blood-based 1,3 β-D-glucan test used to diagnose PCP lacks specificity for *P*. *jirovecii* (11, 12). PCR-based assays for PCP can be more rapid, sensitive, and specific than staining procedures, but also primarily rely on BAL specimens and can detect *P*. *jirovecii* colonization events (*P*. *jirovecii* detected without pneumonia or with pneumonia caused by another pathogen). This can reduce their diagnostic value (13), since PCR values can vary widely in infected individuals, preventing the use of a universal threshold for PCP diagnosis (1, 14, 15). PCR tests have also been used to detect *P*. *jirovecii* DNA in oral wash, induced sputum, and serum specimens, but these tests have variable sensitivity and may also detect colonization events (7, 16–19). There is, therefore, still an urgent need for PCP diagnostics that use less invasive specimen types to provide rapid and accurate results that can guide treatment decisions.

Current PCR tests that target *P*. *jirovecii* genomic DNA can also detect *P*. *jirovecii* colonization events; however, there is no accepted threshold to distinguish colonization from active infection (1, 14, 20). We hypothesized that assays that detect and quantify mRNA transcripts that distinguish the troph and ascus life stages of *P*. *jirovecii*, rather than overall pathogen abundance, could improve specific detection of active infection, since each stage exhibits distinct metabolic activity and behavior during colonization and active infection (21, 22). More sensitive assays may be required to detect such transcripts, however, particularly in less invasive samples where *Pneumocystis* mRNA may be less abundant or rapidly degraded by environmental hydrolases.

CRISPR reactions employed to enhance the sensitivity and specificity of nucleic acid amplification assays (23–25) have been applied to diagnose viral, bacterial, and fungal infections in minimally invasive sample types including blood, saliva, nasal swabs, and urine (25). Such approaches can substantially improve assay sensitivity and specificity, since target amplification and detection relies on specific binding of a reverse transcription (RT) primer (for an RNA target), PCR amplification primers, and a guide RNA sequence that mediates the binding and *trans*-cleavage activity of a target-specific CRISPR Cas complex that can be employed to cleave a quenched reporter nucleotide and amplify the assay readout signal (23, 26). We therefore developed RT-PCR CRISPR Cas12a assays to sensitively and specifically detect *Pneumocystis* mRNAs that differentially overexpressed in the troph and ascus stages of *P*. *jirovecii* and *P*. *murina*, a closely related species that causes fungal pneumonia in mice (21).

Here, we describe the development and characterization of these assays and their performance to detect these stage-selective mRNA targets in serum and BAL samples of *P*. *murina–*infected mice and oropharyngeal swab in infants with *P*. jirovecii infection, and serum and BAL specimens of cohorts of adult patients with PCP. Our results detected a differential increased expression of the troph versus ascus marker in immunocompromised *Rag2^–/–^* mice at increased risk for active infection versus WT mice. Similarly, we observed that the *P*. *jirovecii* troph marker exhibited greater specificity for adults and infants diagnosed with PCP, although both markers were overexpressed in these cases, and there was a clear signal separation in individuals diagnosed with active PCP and those without active disease. These results suggest that similar assays could be employed with oropharyngeal swabs and serum to improve the diagnosis and monitoring of PCP cases required to improve patient outcomes.

## Results

### Study design for RT-PCR CRISPR clinical validation.

RT-PCR CRISPR assays were developed and used to blindly analyze 107 retrospectively collected oropharyngeal swab samples obtained from the PERCH cohort (1), an international case-control study designed to analyze the incidence of pathogens that cause pneumonia in infants (Supplemental Table 1; supplemental material available online with this article; https://doi.org/10.1172/JCI177241DS1). This study examined samples collected from children aged 1–59 months who were admitted to the hospital with severe pneumonia and age-matched healthy controls from the same general communities, and used quantitative PCR to detect the *P*. *jirovecii* gene *mtLSU* in extracted nucleic acid samples at a threshold of greater than 1 × 10^4^ copies/mL as a classifier for active disease. CRISPR and RT-qPCR assay sensitivity and specificity results were calculated against the corresponding PERCH study *mtLSU* qPCR swab results. RT-PCR CRISPR assays were also employed to blindly evaluate 32 BAL samples from 12 individuals with PCP infection and 20 individuals without *P*. *jirovecii* infection, using residual BAL specimens from patients who had PCP-positive pneumonia (qPCR-positive for *P*. *jirovecii mtLSU* DNA) or from patients who were PCP negative undergoing clinical surveillance after lung transplant or for other conditions (Supplemental Table 2).

**Figure 1:**
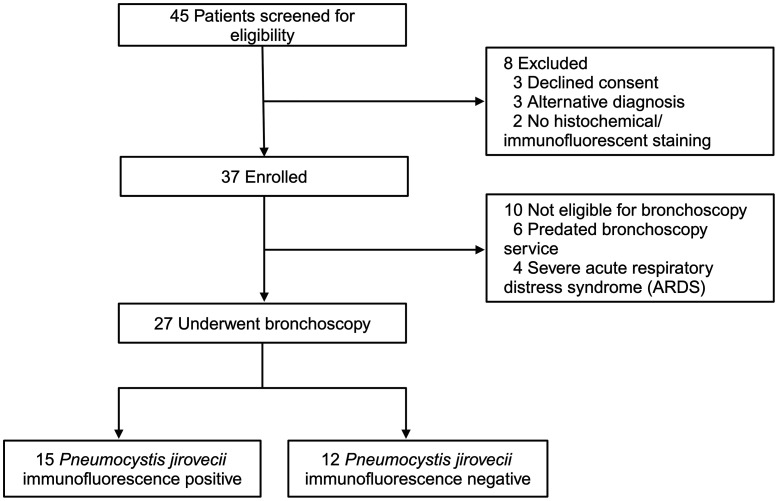
Study Participants from Cape Town, South Africa. After enrollment and screening, RT-PCR CRISPR was evaluated by blind analysis of BAL and serum from a cohort of adult patients from South Africa who were 27 HIV positive with and without PCP confirmed by *Pneumocystis jirovecii* immunofluorescence. Study participants were enrolled with dyspnea and hypoxemia (sO_2_ ≤ 94% or PaO_2_≤ 10kPa) and an abnormal chest X-ray. BAL and serum were obtained from patients at baseline before treatment initiation, and diagnosis was achieved from collected BAL specimens using the *P*. *jirovecii* immunofluorescence assay.

To assess the potential for blood-based PCP diagnosis, CRISPR and RT-qPCR assays were used to blindly analyze matched BAL and serum samples from a prospective cohort of 27 adult patients with HIV with suspected PCP who were enrolled in an observational cohort study at Khayelitsha District Hospital in Cape Town, South Africa (Figure 1). Study participants, who had dyspnea and hypoxemia (sO_2_ ≤ 94% or PaO_2_ ≤ 10kPa) with an abnormal chest X-ray, were provided with PCP treatment and underwent BAL collection to confirm PCP using a *P*. *jirovecii* immunofluorescence assay (IFA) and had serum collected at the same time.

### Development and optimization of CRISPR-enhanced RT-PCR assays for two P. murina mRNA targets.

**Figure 2:**
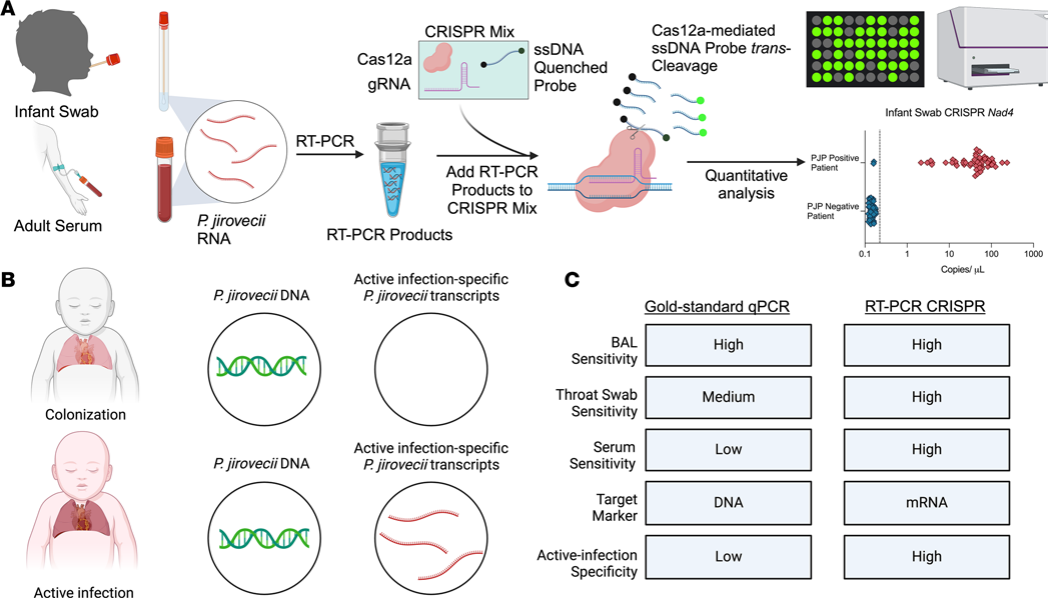
Overview of the RT-PCR CRISPR assay workflow for *P*. *jirovecii* diagnosis. (**A**) RNA isolates from oropharyngeal swab or serum specimens were subjected to RT-PCR to amplify a target mRNA differentially expressed in the fungal trophic form required for active infection. These amplicons were recognized by a Cas12a/gRNA complex that cleaved and derepressed a quenched fluorescent probe in proportion to amplicon abundance. (**B**) DNA and mRNA phenotypes expected in children with *P*. *jirovecii* colonization and infection events and (**C**) characteristics of conventional qPCR and proposed RT-PCR CRISPR assays for *P*. *jirovecii* infection.

*Pneumocystis*-derived biomarkers that distinguish replicating troph and nonreplicating ascus spores could permit development of assays that distinguish *Pneumocystis* infection from colonization to guide treatment decisions (Figure 2). Since we previously reported that *P*. *murina* serine protease (*Sp*) and 1,3-β glucan synthase subunit (*Gsc1*) mRNA transcripts are differentially upregulated in its troph and ascus stages, we hypothesized that RT-PCR CRISPR-Cas12a assays might have the sensitivity necessary to detect them in serum to permit minimally invasive diagnosis. We used an in silico approach to identify primer pairs and gRNAs to amplify and detect target sequences within these mRNAs (Supplemental Table 3).

RT-PCR conditions for these mRNA targets were optimized by analyzing the CRISPR signal produced when their amplicons were generated over a range of annealing temperatures with cDNA generated from lung tissue homogenates of *P*. *murina*–infected mice, as previously described (21). CRISPR signal-to-noise ratios defined by the signal generated with and without input template (Supplemental Figure 1, A and B) identified optimum annealing temperatures for *Sp* and *Gsc1* amplification (57.5°C and 59.9°C) that were used in all further analyses. Subsequent analyses identified the determined reporter concentration (667 pM) that produced the highest signal-to-noise ratio for the least amount of input probe (Supplemental Figure 1, C and D), and the Cas12a/gRNA concentration (67 pM) that yielded optimum signal kinetics for the amount of input Cas12a and gRNA (Supplemental Figure 1, E and F). No substantial signal increases were observed in the absence of input template, consistent with minimal reporter degradation.

Linearity and limit of detection (LoD) values for these optimized *Sp* and *Gsc1* RT-PCR CRISPR assays were then determined using serial dilutions of synthetic *Sp* or *Gsc1* DNA fragments spiked into healthy serum (1 × 10^–1^ to 1 × 10^6^ copies/μL) (Supplemental Figure 2, A and B). These *Sp* and *Gsc1* assays detected positive signals in serum concentration standards spiked with 0.3 and 1 copies/μL, respectively and had strong linear correlations with the spiked-in target amount (R^2^ values of 0.990 and 0.983) from their LoDs to the highest analyzed target concentration (1 × 10^4^ copies/ μL) (Supplemental Figure 2, C and D). *Sp* and *Gsc1* assay signal also demonstrated strong species specificity since positive signal was not detected when these assays were used to analyze genomic RNA or DNA of an array of common viral and microbial respiratory pathogens, including the related human pathogen *P*. *jirovecii* (Supplemental Figure 2, E and F).

### Sp and Gsc1 detection in BAL and serum of P. murina–infected WT and Rag2^–/–^ mice.

**Figure 3:**
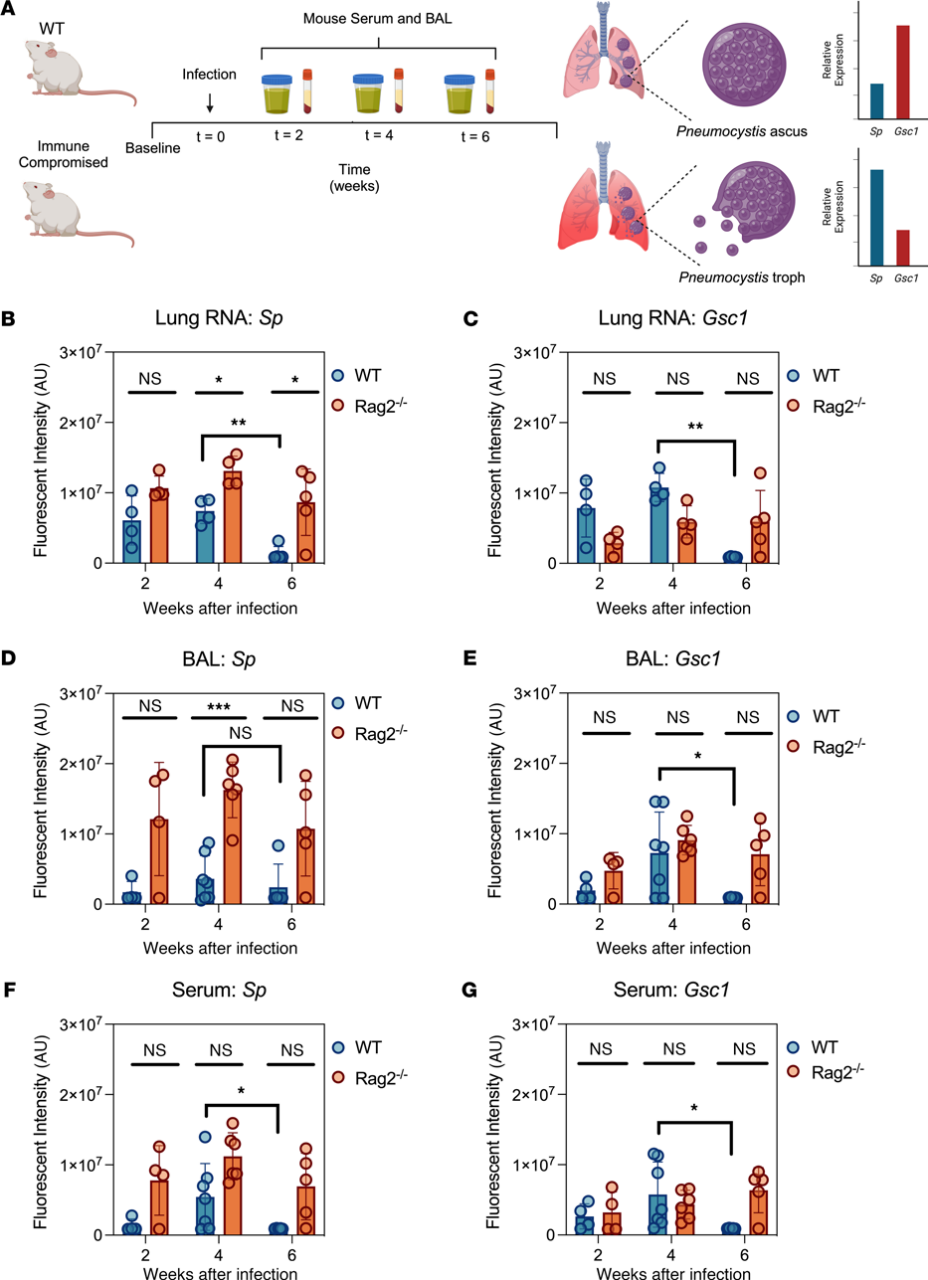
*Sp* and *Gsc1* assay performance in serial BAL and serum from *P*. *murina–*infected mice. (**A**) Scheme showing mouse infection and sampling time course with analysis of *P*. *murina* ascus- and trophic-life form transcripts *Sp* and *Gsc1*. *Sp* and *Gsc1* assay signal in mouse (**B** and **C**) lung RNA, (**D** and **E**) BAL and (**F** and **G**) serum at 2-, 4-, and 6-weeks after inoculation with *P*. *murina*. Graphs indicate mean ± SD values of triplicate samples. *P < 0.05, **P < 0.01, ***P < 0.001, by 2-sample Welch’s *t* test corrected for multiple comparisons by the Holm-Šidák method (WT versus Rag2^–/–^) or performed without correction (4 versus 6 weeks after infection).

*Sp* and *Gsc1* RT-PCR CRISPR assays were used to analyze lung tissue, BAL, and serum specimens collected from C57BL6/J WT and immunocompromised (*Rag2*^–/–^) mice sacrificed 2-, 4-, and 6-weeks after inoculation with *P*. *murina* (Figure 3A), as this model reflects critical aspects of human disease (27, 28). Lung tissue *Sp* mRNA expression was higher in *Rag2^–/–^* than WT mice, and *Gsc1* mRNA expression was higher in the lungs of WT than *Rag2^–/–^* mice (Figure 3, B and C). Lung tissue *Sp* and *Gsc1* signal did not vary over time in *Rag2^–/–^* mice, but both significantly decreased at 6 weeks after inoculation in the WT mice, potentially indicating infection clearance. *Sp* and *Gsc1* signal was less reliably detected in the BAL and serum samples of these mice (Figure 3, D–G), particularly the WT mice. *Sp* and *Gsc1* signal was consistently detected in *Rag2^–/–^* mouse BAL and serum specimens at 4 weeks after inoculation, but signal for both targets was more variable in the matching WT mouse samples and in samples collected at 2 weeks after inoculation in both groups. *Sp* and *Gsc1* signals tended to be greater in *Rag2^–/–^* mouse BAL versus serum specimens, and *Sp* signal tended to be consistently greater than *Gsc1* signal throughout infection, consistent with a reduced ability of the *Rag2^–/–^* mice to suppress their *P*. *murina* infections, as neither difference was detected in the WT mouse samples. *Sp*-positive *Rag2^–/–^* mouse BAL and serum samples also tended to be *Gsc1*-positive by week 2 after inoculation, with double-positive results detected in all *Rag2^–/–^* mouse BAL and serum samples by week 4 after inoculation. By contrast, BAL and serum samples of the WT mice tended to be *Sp*-negative and *Gsc1*-negative at week 2 after inoculation, sporadically positive for both markers at week 4 after inoculation, and mostly negative for both markers at week 6 after inoculation, consistent with greater containment of their *P*. *murina* infections.

### Development and optimization of CRISPR-enhanced RT-PCR assays for P. jirovecii RNA targets.

**Figure 4:**
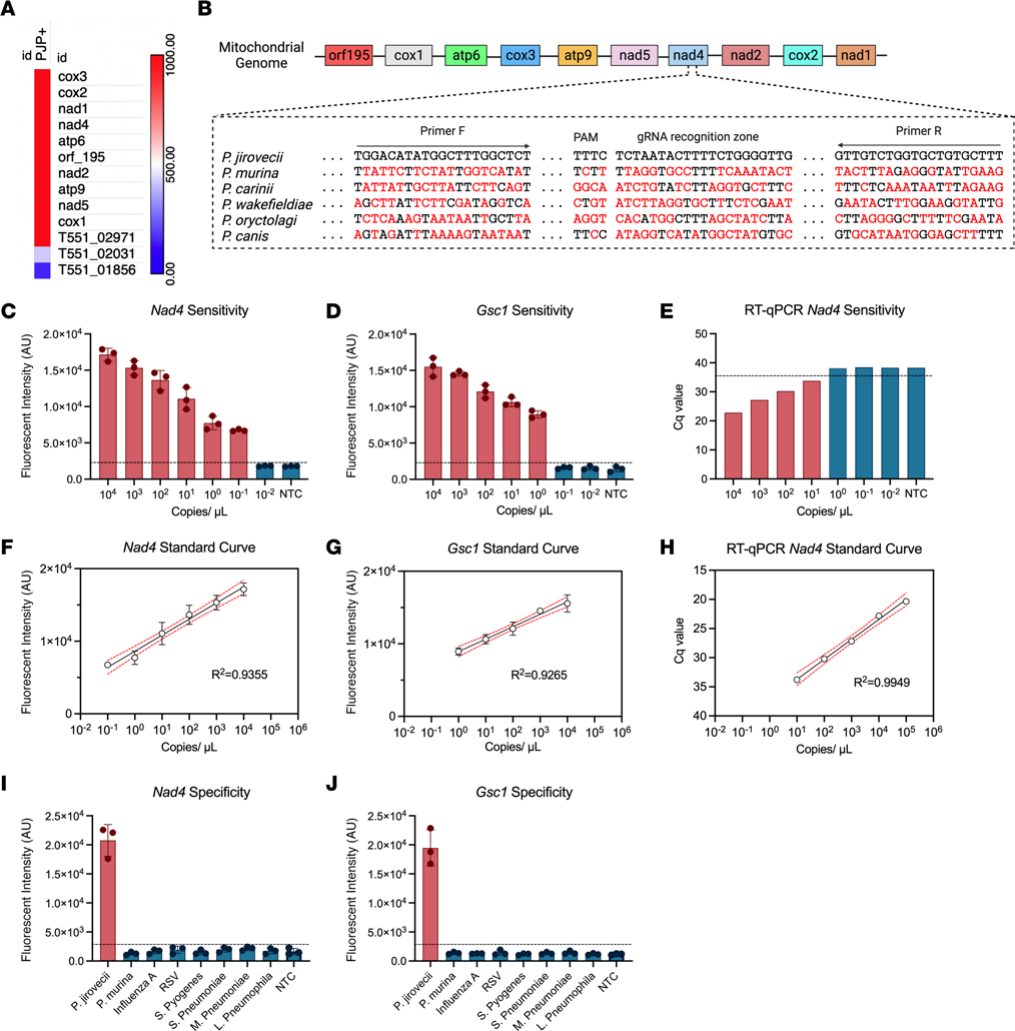
Characterization of *Nad4* and *Gsc1* assay performance in spiked samples. (**A**) Ranked list of the most abundant and differentially detected *P*. *jirovecii* RNAs identified by sequencing of BAL samples of two patients who were positive for *P*. *jirovecii* after subtractive hybridization to remove host-derived RNA transcripts. (**B**) Genomic organization of enriched *P*. *jirovecii* mitochondrial genes and alignment of the *P*. *jirovecii Nad4* primer and gRNA sequences with corresponding sequence regions of other *Pneumocystis* species (red text denotes sequence mismatches). LoD analyses for the (**C**) *Nad4* and (**D**) *Gsc1* CRISPR assays and (**E**) a matching *Nad4* RT-qPCR assay, and the linear detection range data for the (**F**) *Nad4*, (**G**) *Gsc1* CRISPR assays, and for (**H**) RT-qPCR *Nad4*. Species specificity of the *P*. *jirovecii* (**I**) *Nad4* and (**J**) *Gsc1* assays when analyzing samples spiked with corresponding sequences from other respiratory pathogens. NTC, no template control. Graphs indicate mean ± SD values of triplicate analyses. Standard curve graphs indicate the linear regression line of the data, its 95% CI, and Pearson coefficient.

We next translated this approach to detect troph and ascus targets of *P*. *jirovecii*, as this human pathogen is closely related to *P*. *murina*. However, while a *P*. *jirovecii*–specific *Gsc1* primer and gRNA set produced strong signal, those generated for the *Sp* homolog of *P*. *jirovecii* did not produce detectable signal (data not shown), likely due to low confidence in *P*. *jirovecii* *Sp* sequence data or polymorphisms. We therefore, instead, identified *P*. *jirovecii* RNAs that were differentially expressed and abundantly detected in a RNA-seq dataset of BAL specimens from 2 patients who were immunocompromised and diagnosed with *P*. *jirovecii* infections (29). Similar to previous work indicating that mitochondrial transcripts are enriched in troph-derived *P*. *murina* RNA, *P*. *jirovecii* mitochondrial RNAs were the most abundant differentially enriched transcripts detected in these samples (Figure 4, A and B), consistent with a previous study indicating that the trophic form of *P*. *jirovecii* plays a dominant role in pulmonary infections and that troph-derived *P*. *murina* RNA is enriched for mitochondrial RNA transcripts (21). NADH-ubiquinone oxidoreductase chain 4 (*Nad4*) was selected for further analysis since primers to this RNA amplified a region containing a candidate gRNA sequence with a conserved protospacer adjacent motif (PAM) site required for efficient Cas12a target recognition and cleavage activity. These primers and gRNA sequences were designed to avoid known *Nad4* SNPs that might affect their binding and detection and lack substantial homology with corresponding *Nad4* sequences of other *Pneumocystis* species.

CRISPR signal-to-noise ratio analyses determined that annealing temperature (59.9°C) and reporter probe and Cas12a/gRNA complex concentration (67 pM and 67pM) conditions for optimal *P*. *jirovecii* *Nad4* and *Gsc1* RT-PCR Cas12a reactions were similar to those identified for the *P*. *murina* assays (Supplemental Figure 3). The *P*. *jirovecii* *Nad4* and *Gsc1* assays had LoD values (0.1 and 1 copies/μL) (Figure 4, C and D) that closely matched those of the corresponding *P*. *murina* assays, while the LoD value of the equivalent *Nad4* RT-PCR assay was 100× greater than the *Nad4* RT-PCR CRISPR assay (10 copies/μL) (Figure 4E). These RT-PCR CRISPR assays and RT-qPCR revealed strong linear correlations between signal and spiked-in target (R^2^ values of 0.936, 0.927, 0.99) from their individual LoDs to the highest analyzed target concentration (10^4^ copies/μL) (Figure 4, F–H). Finally, both RT-PCR CRISPR assays demonstrated strong species specificity, since strong positive signal was detected in the *P*. *jirovecii*–positive control sample, while negative control samples containing corresponding DNA regions from other respiratory pathogens, including *P*. *murina*, did not produce signal greater than that detected in the nontemplate control sample (Figure 4, I and J).

### P. jirovecii Nad4 and Gsc1 assay performance with patient BAL and oropharyngeal swab specimens.

**Figure 5:**
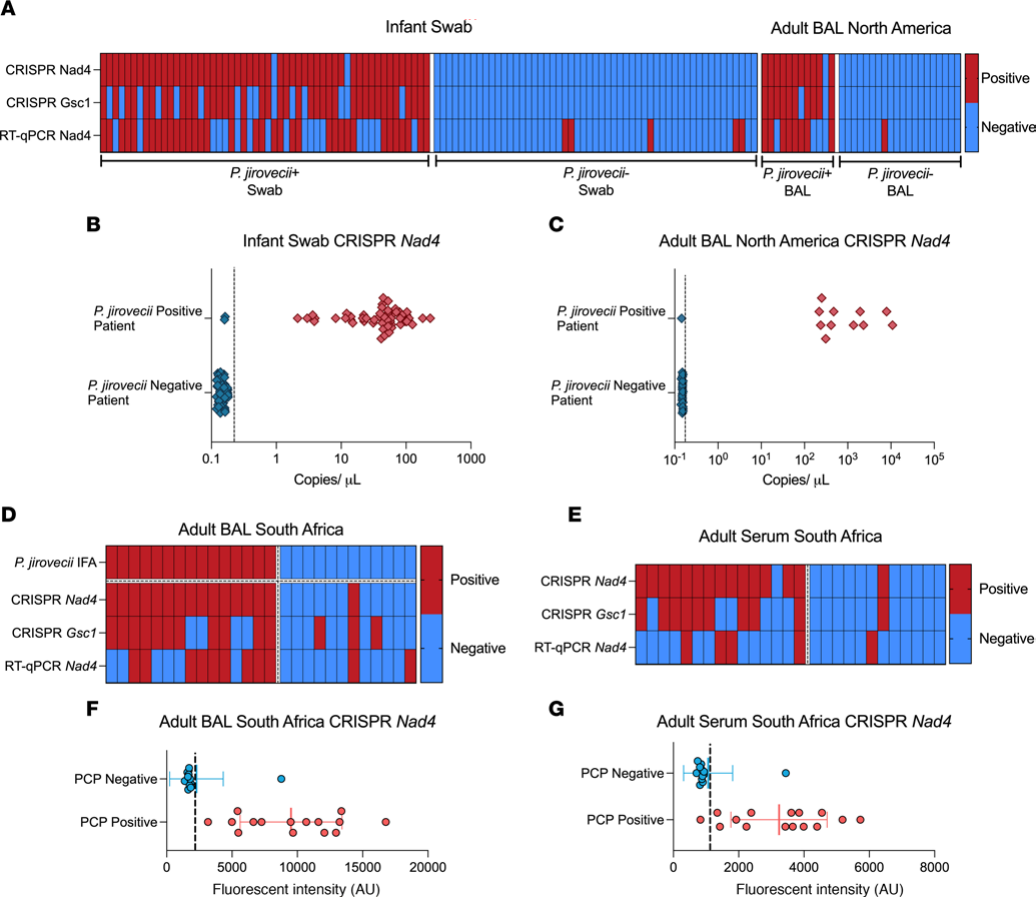
Characterization of *Nad4* assay performance with infant oropharyngeal swab and adult BAL samples. (**A**) Heatmap of CRISPR and RT-qPCR assay positive (red) and negative (blue) results for *Nad4* and *Gsc1* in infant oropharyngeal swab and adult BAL samples from patients with and without *P*. *jirovecii* infection. *Nad4* levels detected in (**B**) infant oropharyngeal swab and (**C**) adult BAL samples from North America, where positive signal was defined as signal that exceeded a threshold of the mean plus 3 times the SD of triplicate NTC samples (vertical dashed lines). (**D** and **E**) Heatmap of CRISPR and RT-qPCR assay positive (red) and negative (blue) results for *Nad4* and *Gsc1* in adult BAL and serum samples from PCP-positive and -negative cases determined by immunofluorescence assay (IFA). *Nad4* levels detected in (**F**) adult BAL and (**G**) adult serum samples from patients in South Africa, where positive signal was defined as signal that exceeded a threshold of the mean plus 3 times the SD of triplicate NTC samples (vertical dashed lines).

For oropharyngeal swab analysis, tested samples were primarily from infants less than 12 months of age (2 children were older than 12 months of age). *Nad4* and *Gsc1* signal thresholds distinguished infants with and without *P*. *jirovecii* infections with 96.3% and 72.2% sensitivity and 100% specificity (Figure 5, A and B, Supplemental Figure 4A, and Table 1), while the *Nad4* RT-qPCR threshold for positive signal had 66.7% diagnostic sensitivity and 90.6% specificity. Similarly, an analysis of adult BAL specimens (12 PCP and 20 non-PCP cases, including 1 patient who was HIV-positive with PCP), detected PCP cases with 91.7% and 83.3% clinical sensitivity and 100.0% specificity (Figure 5C, Supplemental Figure 4B, and Table 2), while *Nad4* RT-qPCR results had 66.7% diagnostic sensitivity and 94.7% specificity. CRISPR *Nad4* and *Gsc1* assay results had better overall classification performance than RT-qPCR *Nad4* assay results to distinguish cases and controls in infant swab and adult BAL sample cohorts when these results were evaluated in ROC curve analyses (Supplemental Figure 5).

### P. jirovecii Nad4 and Gsc1 assay performance with matched patient BAL and serum specimens.

CRISPR *Nad4* signal in BAL specimens from South Africa distinguished patients who were PCP positive and PCP negative with 100% sensitivity and 91.7% specificity, exceeding CRISPR *Gsc1* (73.3% sensitivity / 75.0% specificity) and RT-qPCR *Nad4* (60.0% sensitivity / 83.3% sensitivity) diagnostic performance (Figure 5D, Table 3, and Supplemental Figure 6A). CRISPR *Nad4* and *Gsc1* results for serum identified patients who were PCP positive with 93.3% and 60.0% sensitivity, respectively, and 91.7% specificity, which also exceeded the performance (26.7% sensitivity / 91.7% specificity) of the matching RT-qPCR *Nad4* results (Figure 5E, Table 4, and Supplemental 6B). *Nad4* levels detected in these samples demonstrated higher MFI in BAL versus serum specimens (Figure 5, F and G). CRISPR *Nad4* assay results from adult BAL and serum samples also had better performance to distinguish adult PCP and non-PCP cases than matching *Gsc1* assay results when both were evaluated by ROC curve analysis (Supplemental Figure 7).

## Discussion

New PCP diagnostic tests that employ minimally invasive or noninvasive specimens and distinguish infection from colonization are needed to improve PCP diagnosis, since collecting diagnostic BAL specimens can delay diagnosis, and rapid and sensitive PCR-based assays for *P*. *jirovecii* genomic DNA lack accepted thresholds to distinguish colonization and infection. Herein we describe the development of an ultrasensitive RT-PCR CRISPR assay to detect mRNA targets enriched in the replicating *Pneumocystis* trophic stage associated with active infection and the nonreplicating ascus stage, and the performance of these assays to detect *Pneumocystis* infections in a mouse model of *P*. *murina* pneumonia and in adult and infant cohorts of *P*. *jirovecii* infection using BAL specimens or less invasive samples, including serum and oropharyngeal swabs. CRISPR-mediated signal enhancement was necessary to achieve robust diagnostic sensitivity as it markedly increased the performance of RT-qPCR for *Nad4* mRNA when applied to analyze infant oropharyngeal swab (96.3% versus 66.7%) and adult serum (93.3% versus 26.7%) samples and adult BAL specimens obtained from North American (91.7% versus 66.7%) and South African patient cohorts (100% versus 60.0%).

We have previously used similar CRISPR-Cas12a assay approaches to diagnose respiratory infections caused by other pathogens, including SARS-CoV-2 and *Mycobacterium tuberculosis*, using minimally or noninvasive sample types such as blood and saliva (24, 30, 31), while another group has used CRISPR to diagnose PCP (32). This group used a CRISPR Cas13-based assay approach to detect a *P*. *jirovecii* mitochondrial large subunit ribosomal RNA target in RNA extracts of patient BAL specimens after transcription-mediated amplification. Notably, this approach differs from ours in at least one key aspect since its target was selected for its abundance, repetitive sequence, and frequent citation, not for its ability to distinguish the replicative troph and nonreplicative ascus stages of *P*. *jirovecii* or colonization from infection. This Cas13 assay yielded a higher limit of detection (2 versus 0.1 copies/μL) and lower sensitivity estimates (78.9% versus 91.7% and 100%) with BAL specimens than our Cas12a assay, but achieved similar diagnostic specificity (97.7% versus 100% and 91.7%). No alternate samples were analyzed in this Cas13-based study, however, preventing further comparisons.

Infant swab samples analyzed in this study were obtained from the case-control PERCH study, which used a quantitative multiplex PCR assay to examine causes of severe pneumonia in children aged 1–59 months who were hospitalized with severe pneumonia and age-matched children in a healthy control group from the same general population (1). The PERCH study analyzed oropharyngeal swab and induced sputum specimens from these infants and observed high agreement (94.6%) between results of these specimens (33), supporting the potential utility of swab results for the diagnosis of *P*. *jirovecii* infections in this cohort. However, induced sputum results for individual patients were not available for use as the reference standard in our analysis.

The PERCH study detected *P*. *jirovecii* DNA in oropharyngeal samples collected from cases and controls at similar frequency, likely due to high rates of pulmonary colonization in the healthy control group. Other studies have established thresholds for PCP diagnosis to address this problem, but these values vary among studies and there is no standard threshold to distinguish infection from colonization (14, 15, 34, 35). One study has reported that PCR analysis of a single versus multicopy gene can improve specificity for infection versus colonization events, although this may also reduce assay sensitivity (36). Our results indicate that CRISPR-mediated *Nad4* RNA detection could address this issue, since *Nad4* signal was not detected above background in specimens of most individuals not diagnosed with PCP, but had high diagnostic sensitivity (91.7%–100%) for infants infected with *P*. *jirovecii* and adults with PCP. We were not able to directly compare the results from BAL and oropharyngeal swabs in this study since both sample types were not available from the infant or adult cohorts.

We detected elevated levels of the troph marker *Sp* in serum and BAL samples of *Rag2^–/–^* versus WT mice inoculated with *P*. *murina*, consistent with prior reports that *Rag2^–/–^* mice have higher troph life-form burden during active *P*. *murina* infection (21). Future mouse model experiments should investigate changes in the relative abundance of *P*. *murina* troph and ascus stages during active infection initiation, colonization, and reactivation, and potentially animal-to-animal versus environmental transmission and other important questions. For example, specific depletion of asci by treating *P*. *murina–*infected mice with echinocandins could validate the troph-specific expression of *Sp* (and the *P*. *murina* *Nad4* homolog) (21, 37). Serum could be a less invasive option than BAL for PCP diagnosis, although it is easier to obtain oropharyngeal swabs from infants than serum. Other studies have analyzed *P*. *jirovecii* cell-free DNA using PCR in human serum samples with variable sensitivity (50%–100%), likely due to dilute concentration of cell-free DNA targets, and one of these studies had a substantial drop in specificity when testing serum from healthy blood donors (100%) and patients with HIV (71%) possibly due to colonization detection (9, 16, 38). We observe a slight decrease in sensitivity when testing serum versus BAL (93.3% versus 100.0%), although specificity did not differ for these sample types (91.7%). Notably, the single false-positive sample detected had positive CRISPR *Nad4* results for their matching BAL and serum specimens. However, additional information was not available to evaluate whether this patient was a missed PCP-positive case, had *P*. *jirovecii* colonization, or was accurately assessed as a true negative.

*Pneumocystis* cell–free RNA was less frequently detected in serum versus BAL or lung tissue specimens of the *P*. *murina*-infected mice, but signal for the troph-enriched *Sp* target was consistently lower than Gsc1 signal in all specimen types of the WT versus *Rag2^–/–^* mice, consistent with reduced ability of the *Rag2^–/–^* mice to suppress their *P*. *murina* infections. *Nad4* was selected as a *P*. *jirovecii* troph marker since its elevated expression is consistent with increased metabolic activity of replicating *P*. *jirovecii* trophs, and it revealed greater diagnostic sensitivity for *P*. *jirovecii* infection than the ascus marker *Gsc1* when both were analyzed in oropharyngeal swab (96.3% versus 72.2%), BAL (91.7% and 100% versus 83.3% and 73.3%), or serum (93.3% versus 60.0%) specimens. However, the diagnostic performance of *Gsc1* suggests that sensitive detection of any *P*. *jirovecii* RNA target may enable PCP diagnosis, given that rapid RNA degradation expected in diagnostic specimens might limit detection of low burden colonization events.

CRISPR *Nad4* assay results demonstrated high specificity for *P*. *jirovecii* infections in this study, suggesting that studies designed to detect *P*. *jirovecii–*specific RNA or DNA targets in minimally invasive or noninvasive diagnostic specimens from large, well-characterized cohorts could be used to evaluate transmission among close contacts, the incidence of PCP and *P*. *jirovecii* colonization, and its environmental prevalence (39, 40). Further studies could also clarify the clinical impact of *P*. *jirovecii* colonization, which has been linked to COPD severity and is frequently detected during autopsy (41, 42), particularly since the incidence of *Pneumocystis* colonization differs for the general population (approximately 25%), healthcare workers (more than 50%), and individuals who are HIV positive (approximately 69%) (39, 43). Although *P*. *jirovecii* is an obligate pathogen and humans are likely the only reservoir, as *P*. *jirovecii* cannot infect mice, rats, and nonhuman primates (44–47), *P*. *jirovecii* DNA has been detected in pond water and air samples, and the prevalence of *P*. *jirovecii* infection is higher in areas with more green space (48), suggesting *P*. *jirovecii* may survive briefly in an environmental reservoir.

Rapid detection of infants with *P*. *jirovecii* infection using oropharyngeal swab specimens may have substantial clinical relevance, since *P*. *jirovecii* is likely underdiagnosed in the months following birth and has the potential to produce fatal outcomes (49, 50). We propose that a CRISPR-based approach similar to the one described here could be used to analyze the impact of *P*. *jirovecii* in infants with and without pneumonia, following validation studies, as it should permit accurate high-throughput screening of swab specimens routinely collected from infants (51). CRISPR diagnostics are a relatively new technology, but multiple clinical trials are ongoing to diagnose respiratory infections, including pneumonia, using CRISPR-based approaches (52–54). *P*. *jirovecii* point-of-care methods that use PCR-based assays and noninvasive samples are cheaper than tests that employ BAL specimens (55), and assays that use other noninvasive sample types could also improve diagnostic reliability by attenuating or eliminating sample-to-sample variation and dilution errors that affect the analysis of BAL specimens (56, 57). Multiple studies have developed tests that use induced sputum, but these specimens are also subject to sample-to-sample variation, must be analyzed for sample quality when analyzing infant specimens, and cannot be feasibly collected from individuals acting as healthy controls for specificity tests (33, 58). New diagnostics could also be adapted to formats and workflows suitable for analysis by inexpensive point-of-care devices in resource-limited settings, as has been done for CRISPR-based assays that detect other respiratory pathogens (24, 31). Loop-mediated isothermal amplification–based (LAMP-based) assays that use a turbidity readout to detect *P*. *jirovecii* 18s rRNA gene have been reported, but these use invasive BAL specimens or highly variable induced sputum samples and target *P*. *jirovecii* DNA, which increases the likelihood of detecting colonization (59–61).

This study has limitations that may complicate interpretation of its results. For example, *Nad4* assay sensitivity and specificity estimates could be affected by *P*. *jirovecii* colonization, as the presence or absence of active fungus was not confirmed in all samples. However, it is difficult to account for *P*. *jirovecii* colonization, as there is no gold standard for colonization other than histologic analysis of stained BAL samples, which is not realistic in healthy populations. We also cannot evaluate the diagnostic performance of the *Nad4* assay with adult oropharyngeal swab or infant serum specimens, as our cohort lack these samples. The sensitivity and specificity calculations for the adult cohorts in this study are underpowered and subject to substantial variation in future studies. Nevertheless, we believe that similar, validated CRISPR-based assay approaches could improve *P*. *jirovecii* diagnosis and could be incorporated into multiplex CRISPR assays to detect an array of fungal, bacterial, and viral pathogens that cause pneumonia from a single swab specimen.

## Methods

### Sex as a biological variable.

Patient sexes used in this study are specified in Supplemental Tables 1 and 2. Sex was not considered as a biological variable.

### Mice.

Female C57BL/6J WT and Rag2^–/–^ (B6(Cg)-Rag2tm1.1Cgn/J) mice aged 6-to-8 weeks were obtained from The Jackson Laboratory and housed in a pathogen-free environment at the Tulane University Department of Comparative Medicine.

### Mouse P. murina infection procedure.

All mice were infected with *P*. *murina* by oropharyngeal administration as previously published (28, 29, 62). Mice were lightly anesthetized with 2% isoflurane delivered in a box connected to the delivery machine and then fixed vertically on a surgery board, the tongue was extended with forceps, and a 100 μL inoculum containing 2 × 10^5^ *P*. *murina* cysts was administered to the distal part of the oropharynx using a micropipette while gently closing the nose. At 2-, 4-, and 6-weeks after inoculation, mice were euthanized by carbon dioxide inhalation to collect BAL, sera, and lung tissue specimens.

### RNA isolation.

Mouse serum cell-free (cf) RNA was extracted using a Quick-cfDNA/cfRNA Serum & Plasma Kit (Zymo Research, R1072). RNA was extracted from all other samples analyzed using a Quick-RNA Fungal/Bacterial Miniprep Kit (Zymo Research, R2014). All RNA isolates were eluted in 50 μL of DNase/RNase-free water and stored at –80°C until analysis. Positive control and negative control samples were derived from samples from healthy mice or individuals that were then spiked with *P*. *murina* or *P*. *jirovecii* RNA or water, respectively.

### RT-PCR CRISPR analyses.

RT-PCR reactions were generated by adding 5 μL isolated RNA to a mixture containing 10 μL 2× Platinum SuperFi RT-PCR master Mix (Thermo Fisher Scientific, 12594025), 0.2 μL SuperScript IV RT Mix (Thermo Fisher Scientific, 12594025), 1 μL of 10 μM forward primer, 1 μL of 10 μM reverse primer, and 2.8 μL of nuclease free-water. For RT-PCR-CRISPR experiments, 5 μL isolated *P*. *murina* or *P*. *jirovecii* RNA was added as template and water was added for the no template controls. RT-PCR reaction was first incubated at 25°C for 2 minutes and 55°C for 10 minutes to permit cDNA synthesis, and then denatured at 95°C for 5 minutes, subjected to 38 cycles of PCR amplification [95°C for 10 seconds, 60°C for 10 seconds, and 72°C for 15 seconds], and then incubated at 72°C for 5 minutes to permit complete extension of all amplicons. CRISPR reaction mixtures containing 25.48 μL nuclease-free water, 0.01 μL 66.7 μM IDT Lb Cas12a (Integrated DNA Technologies, 10007922), 0.01 μL 100 μM gRNA, 1.5 μL of the 10 μM fluorescent reporter, and 3 μL NEBuffer 2.1 (New England Biolabs, B7202) were supplemented with a 2 μL aliquot the final RT-PCR reaction sample and incubated at 37°C for 15 minutes in the dark in a 96 well Corning half-area opaque plate. CRISPR reactions analyzing *P*. *murina* and *P*. *jirovecii* RT-PCR reaction samples were respectively analyzed using a SpectraMax i3× Multi-Mode Microplate Reader (Molecular Devices) and an Infinite M Plex (Tecan) plate reader, using 485 nM excitation and 525 nM emission settings. Thresholds for positive CRISPR signal in spiked samples and clinical samples were defined as the mean plus 3 times the SD of the signal detected in triplicate no-template control samples.

### Standard curve and LoD analyses.

Serum samples used to generate the standard curves for the *P*. *murina* and *P*. *jirovecii* RT-PCR CRISPR assays were generated by spiking known concentrations of the appropriate *P*. *murina* or *P*. *jirovecii* synthetic gBlock target DNA sequence (*Sp* or *Gsc1* and *Nad4* or *Gsc1*) into healthy mouse serum or swab RNA isolation solution, respectively. These concentration standards were then subjected to 10-fold serial dilutions in serum or swab diluent to generate concentration standards that contained from 1 × 10^–1^ to 1 × 10^6^ copies/μL of these target sequences. These concentration standards were then processed to isolate DNA that was analyzed in RT-PCR CRISPR assays for the appropriate target sequence.

### RT-qPCR.

RT-PCR reactions were performed with SuperScript IV First-Strand Synthesis System kits and random hexamers (Thermo Fisher Scientific) and the resulting cDNA was isolated using AMPure XP Beads (Beckman Coulter, A63880) and 80% ethanol before use in qPCR reactions employing 10 μL SsoAdvanced Universal Probes Supermix (2 ×) (Bio-Rad, 172-5280), 0.9 μL forward primer (20 μM), 0.9 μL reverse primer (20 μM), 0.45 μL probe (20 μM), 5.75 μL nuclease-free water, and 2 μL cDNA template. Reactions were performed by incubating the reactions at 50°C for 2 minutes and 95°C for 10 minutes, and then using 50 cycles of 95°C for 15 seconds and 60°C for 30 seconds for target amplification. Melt curves were performed from 55°C to 95°C with 0.5°C increments after reaction completion to confirm that the reaction amplified a single product with the expected melting temperature profile. Thresholds for positive RT-qPCR signal in spiked samples were defined as the mean plus 3 times the SD of the signal detected in triplicate no-template control samples. Thresholds for positive RT-qPCR signal in clinical samples were determined by ROC curve analyses.

### Clinical sample collection procedures.

Oropharyngeal swabs analyzed in this study were obtained from 107 children between 1 and 59 months (Supplemental Table 2); 54 who were *P*. *jirovecii* infected and 53who were not *P*. *jirovecii* infected. Participants with *P*. *jirovecii* infection enrolled in the PERCH cohort study were judged to be infected with *P*. *jirovecii* if their analyzed swab samples yielded greater than 1 × 10^4^ copies/mL of *mtLSU* DNA when analyzed using a quantitative multiplex PCR assay (FTD Resp-33 kit; Fast-track Diagnostics). Individuals who were uninfected with *P*. *jirovecii* and were controls were age-matched with cases and selected from communities near the study sites. Children were deemed HIV positive if HIV virus was detected in their serum samples or if the child was seropositive for HIV at greater than 12 months of age. Swabs were collected in viral transport medium (universal transport medium [UTM], Copan Diagnostics) and processed to extract nucleic using the NucliSENS easyMAG platform (bioMerieux, Marcyl’Etoile, France) (63). Adult BAL samples collected in Toronto and New Orleans represented residual clinical pathology laboratory samples and were sampled according to clinical guidelines (64). *P*. *jirovecii*-positive and *P*. *jirovecii*-negative BAL specimens were obtained from adult patients with pneumonia or who underwent clinical surveillance following lung transplant or in response to other conditions and whose BAL samples respectively tested positive and negative when analyzed by RealStar *Pneumocystis jirovecii* PCR kit 1.0 (altona Diagnostics). Adult serum and BAL specimens obtained from South Africa were collected as part of an NIHR-funded prospective observational study aimed at describing outcomes and evaluation of noninvasive diagnostic tests for HIV-associated PCP. Consecutive adults with probable (clinical case definition) or definite (immunofluorescent staining on a respiratory sample) HIV-associated PCP were enrolled from a District Hospital in the township of Khayelitsha, Cape Town. Eligible participants underwent bronchoscopy and evaluation for coinfections. Bronchoscopies and BAL sample collections were performed by a respiratory physician using a flexible fiber-optic bronchoscope. Procedures were performed through the oral cavity following local anesthesia (lidocaine 2%) and were supported by cardiopulmonary monitoring (continuous assessment of pulse rate, blood pressure, and oxygen saturation). All BAL samples were obtained from areas of lung infiltration, and if multiple areas were observed, the samples were obtained from the area where the infiltration was most severe.

### RNA-seq.

RNA was extracted with the Trizol method from the BAL cell pellets of 2 patients with Bare Lymphocyte Syndrome who had clinical PCP. Prior to construction of an Illumina total RNA library, DNase-treated RNA was quantitated using a Qubit RNA BR assay kit (Thermo Fisher Scientific: Guide MAN0001987 MP10210, Kit #Q10210). Cytoplasmic, mitochondrial, and bacterial rRNA was removed from 2.5 μg of each sample as indicated by the Illumina RiboZero RNA Removal Kit Reference Guide [(Document # 15066012 v02, ScriptSeq Complete Gold (Epidemiology) Kit #BEP1206 (now obsolete)]. Illumina-compatible cDNA libraries were generated according to the instructions of the TruSeq Stranded Total RNA Sample Preparation Guide (Illumina Document #1000000040499v00, Kit #20020596). All libraries were pooled and denatured following the standard normalization method described by the Illumina Denature and Dilute Libraries Guide for the NextSeq System (Illumina Part #15048776), after which denatured libraries were loaded onto an Illumina NextSeq 550. To determine transcript abundance, FASTQ outputs were aligned to the *Pneumocystis jirovecii* RU7 genome using EdgeR normalization (65).

### Statistics.

Statistical analyses were performed using GraphPad Prism 10 software, where *P* values of less than 0.05 were used to determine statistically significant differences between groups when analyzed by parametric or nonparametric *t* tests according to their data characteristics.

### Study approval.

Adult BAL samples analyzed in this study were obtained from residual deidentified clinical diagnostic specimens using an IRB-approved informed consent process at Ochsner Medical Center – New Orleans (Pro00015109) and University Health Network Toronto (13-7093). Adult BAL and serum specimens from the South African cohort were collected as part of a prospective observational cohort study performed in compliance with a protocol approved by the University of Cape Town Human Research Ethics Committee (HREC 543/2022). The Tulane University IRB reviewed the analysis protocol for the deidentified PERCH oropharyngeal swab samples (protocol 2021-1332) and determined it to be nonhuman-subject research. Mouse model studies were performed in compliance with a protocol approved by the Tulane Institutional Animal Care and Use Committee (protocol 1821). All study participants or parents or guardians of study participants gave written informed consent.

### Data availability.

No original code was generated for this study and all reported data is available from the lead author upon request. Sequencing data for this study is available at GEO accession number GSE289324. Values for all data points in the graphs are reported in the Supporting Data Values file.

## Author contributions

BMY, SH, ATM, RPJL, SW, BN, JKK, and TYH designed the research studies. BMY, DP, GD, CFN, and YA conducted the experiments. BMY, ATM, DP, AS, and CFN acquired the data. BMY, BN, CJL, JKK, and TYH analyzed the data. JKK and TYH provided reagents. BMY, CJL, JKK, and TYH wrote the manuscript. Among the co–first authors, BMY was listed first because he developed the CRISPR assay used in the paper.

## Supplementary Material

Supplemental data

ICMJE disclosure forms

Supporting data values

## Figures and Tables

**Table 2:**
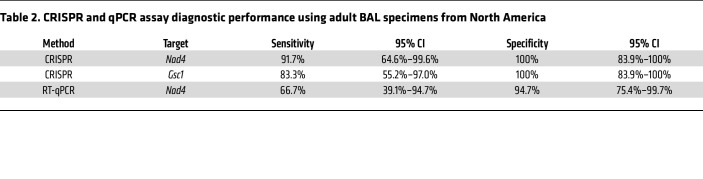
CRISPR and qPCR assay diagnostic performance using adult BAL specimens from North America

**Table 1:**
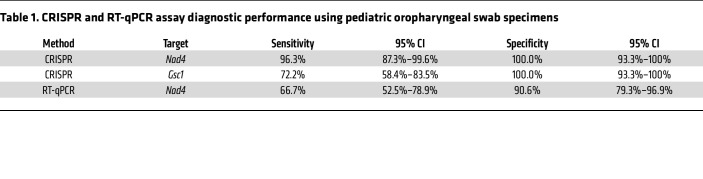
CRISPR and RT-qPCR assay diagnostic performance using pediatric oropharyngeal swab specimens

**Table 3:**
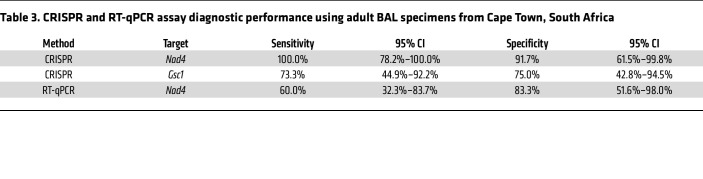
CRISPR and RT-qPCR assay diagnostic performance using adult BAL specimens from Cape Town, South Africa

**Table 4:**
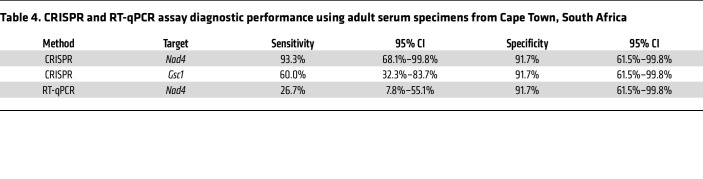
CRISPR and RT-qPCR assay diagnostic performance using adult serum specimens from Cape Town, South Africa

## Version 1 03/03/2025

In-Press Preview

## Version 2 04/15/2025

Electronic publication

## Version 3 08/13/2025

Updated supporting data values figure numbering
