# Supplementary material for: CRISPR-mediated detection of Pneumocystis transcripts in bronchoalveolar, oropharyngeal, and serum specimens for Pneumocystis pneumonia diagnosis
Source: J Clin Invest. 2025 Mar 3;135(8):e177241. doi: 10.1172/JCI177241 (PMC11996908; doi:10.1172/JCI177241)
Supplement: Supplemental data [file jci-135-177241-s010.pdf]

Supplemental Material

CRISPR-mediated detection of *Pneumocystis* transcripts in bronchoalveolar, oropharyngeal, and serum specimens for *Pneumocystis* pneumonia diagnosis

Brady M. Youngquist, Ayanda Trevor Mnguni, Dora Pungan, Rachel PJ Lai, Guixiang Dai, Chun Fai Ng, Amy Samson, Yasmean Abdelgaliel, Christopher J. Lyon, Bo Ning, Shahid Husain, Sean Wasserman, Jay K. Kolls and Tony Y. Hu

Supplementary Figures

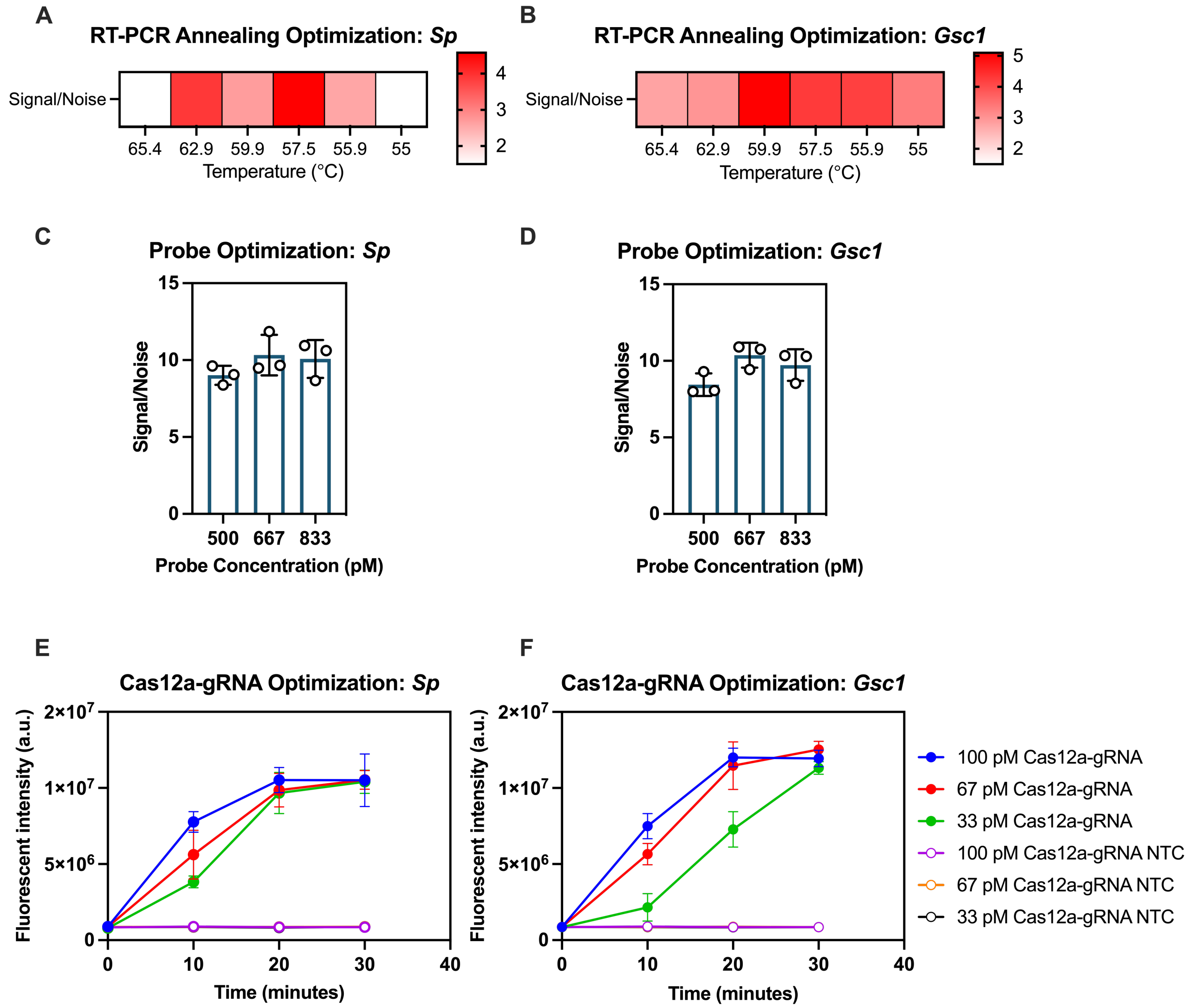

Supplementary Figure 1: Optimization of RT-PCR-CRISPR assays for *P. murina* RNA targets. Signal-to-noise ratios of *Sp* and *Gsc1* RT-PCR CRISPR assays performed with DNA isolated from healthy serum spiked with *Sp* or *Gsc1* PCR amplicons at **(A and B)** the indicated annealing temperatures, with **(C and D)** the listed probe concentration, and **(E and F)** with the indicated Cas12a/gRNA complex concentrations. Signal-to-noise calculated as the ratio of fluorescent intensity for a sample well versus the mean fluorescent intensity in its matching no template control (NTC) wells.

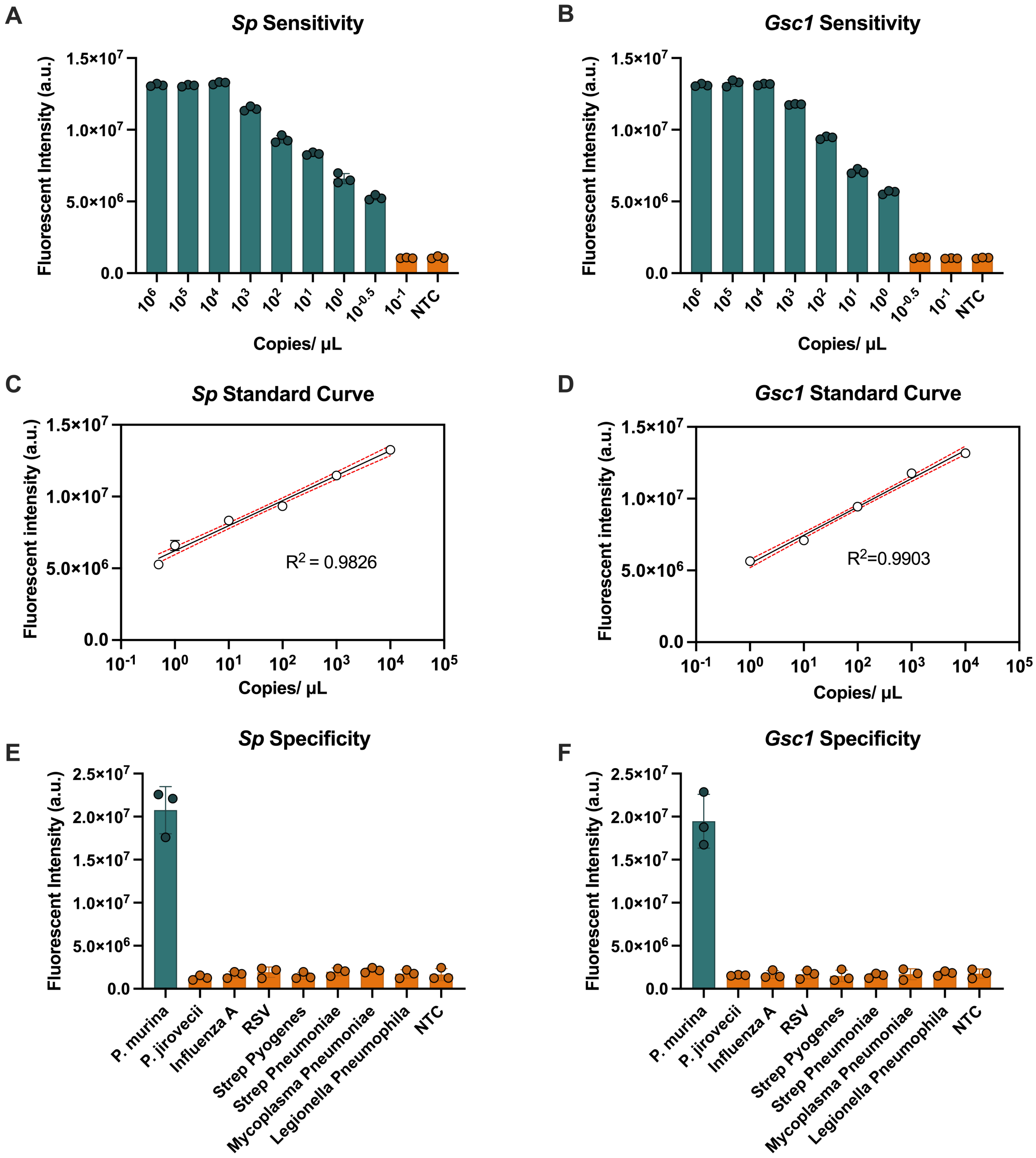

Supplementary Figure 2: Characterization of *Sp* and *Gsc1* assay performance in spiked serum. *Sp* and *Gsc1* assay **(A and B)** limit of detection analysis, **(C and D)** standard curve linear range data, and **(E and F)** specificity results when analyzing serial dilutions of DNA isolated from healthy mouse serum spiked with *Sp* or *Gsc1* PCR amplicons. Standard curve graphs indicate linear regression line, 95% CI, and Pearson coefficient. Sensitivity and Specificity graphs indicate mean ± SD values of triplicate samples.

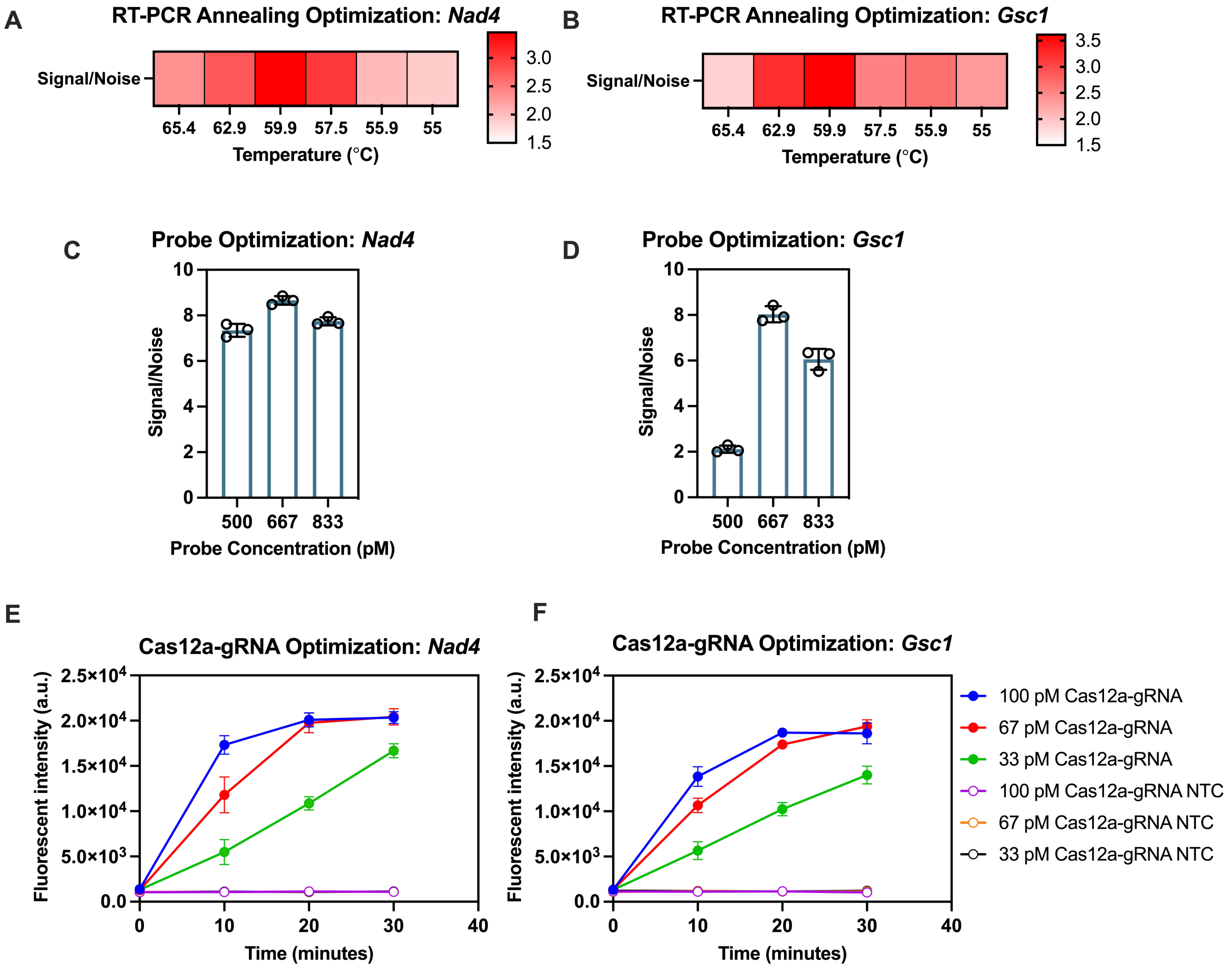

Supplementary Figure 3: Optimization of RT-PCR-CRISPR assays for *P. jirovecii* RNA targets. Signal-to-noise ratios of *Nad4* and *Gsc1* RT-PCR CRISPR assays performed with DNA isolated from healthy serum spiked with *Nad4* or *Gsc1* PCR amplicons at **(A and B)** the indicated annealing temperatures, with **(C and D)** the listed probe concentrations, and **(E and F)** with the indicated Cas12a/gRNA complex concentrations. Signal-to-noise calculated as the ratio of fluorescent intensity for a sample well versus the mean fluorescent intensity in its matching no template control (NTC) wells. Cas12a-gRNA graphs indicate mean ± SD values of triplicate samples.

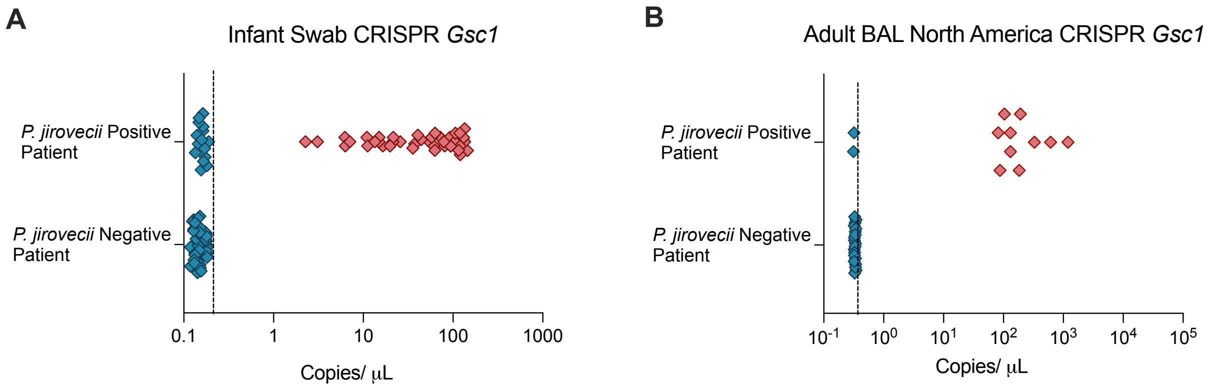

Supplementary Figure 4: *Gsc1* levels from infant swabs and adult BAL samples. *Gsc1* levels detected in **(A)** infant oropharyngeal swab and **(B)** adult BAL samples from North America, where positive signal was defined as signal that exceeded a threshold of the mean plus three times the SD of triplicate NTC samples (vertical dashed lines).

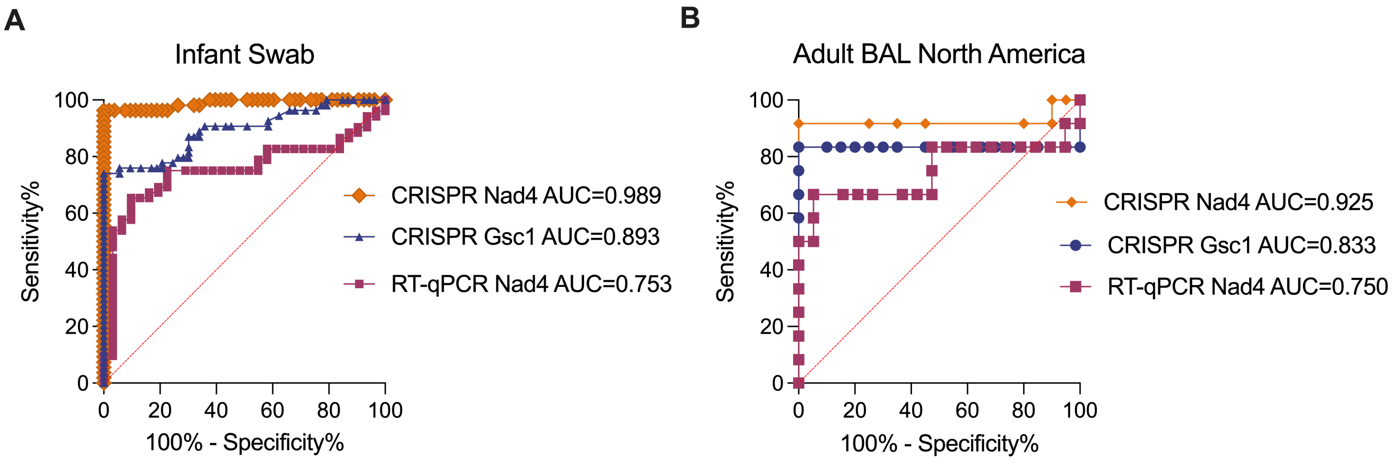

Supplementary Figure 5: ROC curves for infant swab and North American BAL cohorts. Receiver operating characteristic curve results for the ability of CRISPR *Nad4* and *Gsc1*, and RT-qPCR *Nad4* to distinguish **(A)** *P. jirovecii*-infected and -non-infected cases from pediatric oropharyngeal swabs, and **(B)** PCP-positive and PCP-negative cases from North American adult BAL samples.

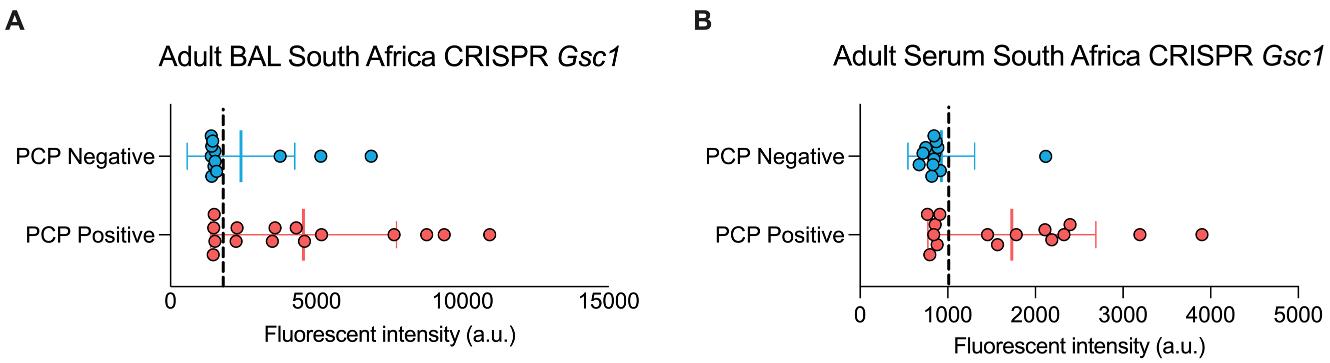

Supplementary Figure 6: *Gsc1* levels from patients in South Africa cohort. *Gsc1* levels detected in adult **(A)** BAL and **(B)** serum samples from patients in South Africa. Positive signal was defined as signal that exceeded a threshold of the mean plus three times the SD of triplicate NTC samples (vertical dashed lines).

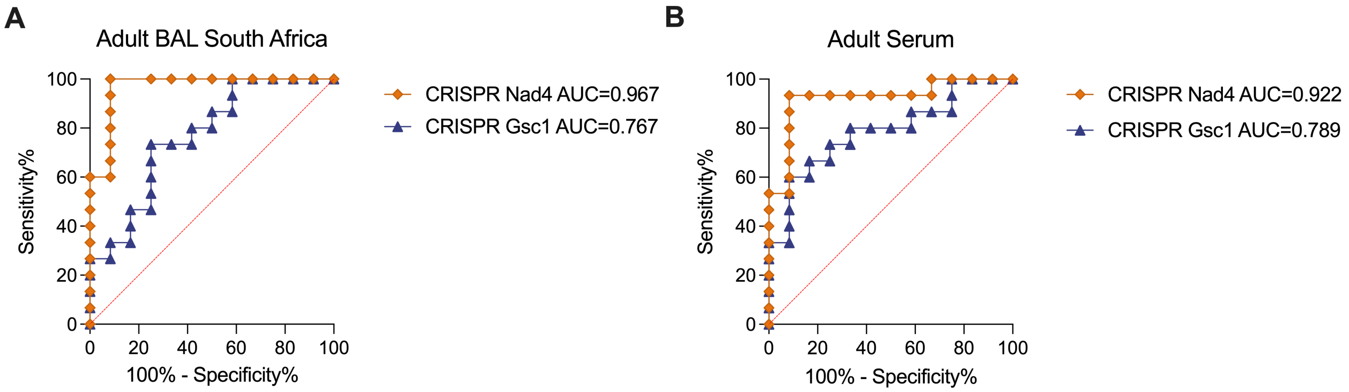

Supplementary Figure 7: ROC curves for South African cohort. Receiver operating characteristic curve results for the ability of CRISPR *Nad4* and *Gsc1* to distinguish PCP-positive and PCP-negative adults from **(A)** BAL and **(B)** serum samples from South Africa.

Supplementary Tables

SUPPLEMENTARY TABLE 1. CLINICAL/DEMOGRAPHIC INFORMATION FOR PERCH INFANTS WITH OROPHARYNGEAL SWABS

SAMPLE #	PJP Status	age (months)	HIV status	Sex	Enrollment site
1	P	5	P	F	Mali
2	P	4	Unknown	M	Mali
3	P	3	N	M	Bangladesh
4	P	2	N	M	Thailand
5	P	3	N	F	Zambia
6	P	2	N	F	Thailand
7	P	3	N	M	Bangladesh
8	P	3	N	M	Bangladesh
9	P	3	Unknown	F	Mali
10	P	3	N	F	Zambia
11	P	3	P	F	Zambia
12	P	2	Unknown	M	Mali
13	P	3	Unknown	M	Mali
14	P	6	N	M	Zambia
15	P	3	P	F	Zambia
16	P	2	N	F	Zambia
17	P	3	P	F	Zambia
18	P	3	P	M	Zambia
19	P	3	N	M	Zambia
20	P	2	N	M	Zambia
21	P	6	N	F	Zambia
22	P	3	N	M	Zambia
23	P	2	N	F	Zambia
24	P	3	N	F	Zambia
25	P	2	N	M	Zambia
26	P	5	Unknown	M	Mali
27	P	3	Unknown	F	Mali
28	P	6	Unknown	M	Mali
29	P	3	P	M	Mali
30	P	4	N	F	Mali
31	P	4	N	F	Mali
32	P	3	Unknown	M	Mali
33	P	3	N	F	Zambia

34	P	4	N	F	Mali
35	P	3	N	F	Mali
36	P	2	Unknown	M	Mali
37	P	3	N	M	Bangladesh
38	P	4	N	M	Bangladesh
39	P	3	N	F	Thailand
40	P	2	N	F	Thailand
41	P	2	N	F	Thailand
42	P	3	N	F	Thailand
43	P	3	P	M	Zambia
44	P	5	N	F	Mali
45	P	3	Unknown	F	Mali
46	P	8	N	M	Bangladesh
47	P	4	N	M	Bangladesh
48	P	3	N	M	Bangladesh
49	P	3	N	F	Bangladesh
50	P	3	N	M	Bangladesh
51	P	2	N	M	Unknown
52	P	4	N	M	Mali
53	P	3	N	M	Mali
54	P	4	Unknown	F	Mali
55	N	1	N	F	Bangladesh
56	N	5	N	F	Zambia
57	N	3	N	M	Bangladesh
58	N	4	N	M	Thailand
59	N	5	Unknown	M	Mali
60	N	3	N	M	Bangladesh
61	N	19	P	M	Zambia
62	N	2	N	M	Zambia
63	N	1	N	M	Zambia
64	N	5	P	F	Zambia
65	N	3	N	M	Mali
66	N	7	Unknown	F	Mali
67	N	4	Unknown	M	Mali
68	N	0	Unknown	M	Mali

69	N	2	N	F	Bangladesh
70	N	1	N	F	Zambia
71	N	2	N	M	Zambia
72	N	1	N	M	Zambia
73	N	1	P	M	Zambia
74	N	1	N	F	Zambia
75	N	3	N	M	Zambia
76	N	5	N	M	Zambia
77	N	3	N	F	Zambia
78	N	4	N	F	Thailand
79	N	5	N	F	Thailand
80	N	4	N	F	Thailand
81	N	2	N	F	Mali
82	N	1	Unknown	M	Mali
83	N	5	N	F	Mali
84	N	3	N	F	Mali
85	N	2	N	M	Mali
86	N	1	N	F	Mali
87	N	2	N	M	Mali
88	N	4	N	F	Thailand
89	N	3	N	M	Thailand
90	N	1	N	F	Thailand
91	N	5	N	M	Thailand
92	N	4	N	F	Thailand
93	N	3	N	M	Thailand
94	N	5	N	M	Thailand
95	N	2	N	M	Bangladesh
96	N	2	N	M	Bangladesh
97	N	3	N	F	Bangladesh
98	N	4	N	M	Bangladesh
99	N	4	N	F	Bangladesh
100	N	5	N	F	Thailand
101	N	5	N	F	Thailand
102	N	4	N	M	Zambia
103	N	10	N	M	Zambia

104	N	21	N	F	Thailand
105	N	11	N	F	Bangladesh
106	N	4	N	M	Bangladesh
107	N	4	N	F	Bangladesh

SUPPLEMENTARY TABLE 2. CLINICAL INFORMATION FOR ADULT PCP SUSPECTS FROM NORTH AMERICA WITH BAL SAMPLES

SAMPLE #	PJP status	Age (years)	HIV status	Sex	Underlying Disease	Non-*P. jirovecii* infections	Enrollment site
1	P	37	Unknown	M	Alcohol-related liver disease, HBV	None	Canada
2	P	30	Unknown	F	COVID-19	*Mycobacterium frankinii, Klebsiella pneumoniae*	Canada
3	P	49	Unknown	M	EGFR exon 19 deletion metastatic lung adenocarcinoma	None	Canada
4	P	37	Unknown	M	Unknown	None	Canada
5	P	46	Unknown	M	Acute myeloid leukemia	None	Canada
6	P	83	Unknown	F	Acute myeloblastic leukemia	None	Canada
7	P	54	Unknown	M	Acute myeloid leukemia	None	Canada
8	P	63	Unknown	F	Unknown	None	Canada
9	P	79	Unknown	M	Acute myeloid leukemia	None	Canada
10	P	75	Unknown	M	Diffuse large B-cell lymphoma	None	Canada
11	P	52	Unknown	F	Multiple myeloma	None	Canada
12	P	31	P	M	Unknown	HIV	United States
13	N	41	Unknown	F	Unknown	None	Canada
14	N	26	Negative	F	Unknown	None	Canada
15	N	69	Negative	F	Unknown	None	Canada
16	N	67	Unknown	M	Unknown	None	Canada
17	N	40	Negative	F	Unknown	*Klebsiella pneumoniae*	Canada
18	N	67	Negative	M	Unknown	None	Canada
19	N	62	Negative	M	Unknown	None	Canada
20	N	71	Unknown	M	Unknown	*Aspergillus fumigatus*	Canada
21	N	62	Negative	M	Unknown	None	Canada
22	N	67	Negative	F	Unknown	*Aspergillus fumigatus*	Canada
23	N	72	Negative	M	Unknown	*Aspergillus fumigatus*	Canada
24	N	75	Negative	F	Unknown	*Trichoderma* species	Canada
25	N	76	Negative	F	Unknown	None	Canada
26	N	25	Negative	M	Unknown	None	Canada
27	N	74	Negative	F	Unknown	None	Canada
28	N	45	Negative	F	Unknown	Influenza A, *Aspergillus fumigatus*	Canada
29	N	78	Negative	M	Unknown	None	Canada
30	N	64	Negative	M	Unknown	None	Canada
31	N	71	Negative	M	Unknown	*Penicillium* species, *Aspergillus niger*	Canada
32	N	76	Unknown	M	Unknown	*Escherichia coli*	Canada

P = positive, N = negative, HBV = hepatitis B virus

Supplementary Table 3 Primers, gRNA, and probe sequences used in this study

Name	Sequence (5' → 3')	Method
RT-PCR primers		
P. murina Sp F	GGTGTTTTAGCCCTAGCAAGC	CRISPR
P. murina Sp R	TGCAGAAAACGAAAGCCCTTG	CRISPR
P. murina Gsc1 F	ATTATGCGCCGGAATATGG	CRISPR
P. murina Gsc1 R	ACTGAAGAGGACGCTGAT	CRISPR
P. jirovecii Nad4 F	AAAGCACAGCACCAGACAAC	CRISPR
P. jirovecii Nad4 R	TGGACATATGGCTTTGGCTCT	CRISPR
P. jirovecii Gsc1 F	TTTAGCATGGTGGACGGGAC	CRISPR
P. jirovecii Gsc1 R	GTCGAGAAGGTCGAAGCCAA	CRISPR
P. jirovecii Nad4 F	CAGACAGTATAGACCAAAGGAGTG	RT-qPCR
P. jirovecii Nad4 R	GTGTTGTTCTTGCGGGTATTG	RT-qPCR
Guide RNAs		
P. murina Sp	UAAUUUCUACUAAGUGUAGAUUGCUGCCUUAAAUGUUGCUUCAC	CRISPR
P. murina Gsc1	UAAUUUCUACUAAGUGUAGAUAGUCAAUUCUUGAACAACCAA	CRISPR
P. jirovecii Nad4	UAAUUUCUACUAAGUGUAGAUUCUAAUACUUUUCUGGGGUUG	CRISPR
P. jirovecii Gsc1	UAAUUUCUACUAAGUGUAGAUUCUGCAAAGUUAUAGAAUUAUC	CRISPR
Probe		
Fluorescent Reporter (all CRISPR targets)	FAM-TTTTTTTTTTTT-BHQ	CRISPR
RT-qPCR Probe *Nad4*	FAM-CGAAGCTTC-ZEN-TGGCAAGATAGGTAACAGA-IABKFQ	RT-qPCR
